# Urinary Cytology: Potential Role in Canine Urinary Tract Infections

**DOI:** 10.3390/vetsci9060304

**Published:** 2022-06-20

**Authors:** Ilaria Lippi, Verena Habermaass, Eleonora Gori, Valentina Virginia Ebani, Alessio Pierini, Veronica Marchetti

**Affiliations:** Department of Veterinary Sciences, Veterinary Teaching Hospital “Mario Modenato”, University of Pisa, 56122 Pisa, Italy; ilaria.lippi@unipi.it (I.L.); verena.habermaass@phd.unipi.it (V.H.); valentina.virginia.ebani@unipi.it (V.V.E.); pierini.alessio2004@gmail.com (A.P.); veronica.marchetti@unipi.it (V.M.)

**Keywords:** UTI, bacteriuria, urinary cytology, unstained urinary sediment, stained urinary sediment, dog

## Abstract

The diagnosis of urinary tract infections (UTIs) requires a concomitant evaluation of clinical signs and urine culture, which is of fundamental to start an appropriate antibiotic treatment. Several factors, such as subclinical bacteriuria or pre-analytical errors, may make the interpretation of urine culture difficult. The aim of the study was to evaluate the association between the finding of neutrophils and bacteria in unstained and stained canine urine sediment and the presence of clinical signs and positive urine culture. Urine samples from 35 dogs with clinical signs of UTI and 55 asymptomatic dogs with risk factors for UTI were prospectively collected by cystocentesis, divided into three aliquots, and submitted for: (1) physical and chemical Dipstick analysis and unstained urinary sediment (casts, crystals, bacteria, leucocytes, cells, parasites); (2) stained urinary sediment (extra/intracellular bacteria, degenerated and non-degenerated neutrophils); (3) qualitative and quantitative urine culture and antimicrobial sensitivity-test. The association between unstained and stained findings of urinary sediment and urine culture was tested. Sensibility, specificity, and positive/negative predictive values in diagnosing positive urine cultures of bacteria at unstained and stained evaluation were compared. Both wet-mount bacteriuria and the cytological presence of intracellular and extracellular bacteria, neutrophils, and degenerated neutrophils were successively associated with positive urine culture (*p* < 0.001). The presence of intracellular bacteria was the only independent predictor of positive urine culture. Total bacterial count did not differ significantly between symptomatic and asymptomatic dogs. Detection of extracellular and intracellular bacteriuria at stained urinary sediment significantly improved the sensibility of predicting positive urine culture. Cytologic evaluation of urinary sediment may be helpful in detecting signs of active inflammation, thus enhancing the clinical relevance of a positive urine culture.

## 1. Introduction

In veterinary medicine, the diagnostic process for urinary tract infections (UTIs) may be challenging, especially for its therapeutic implications and, above all, the correct use of antibiotics. From a One Health perspective, the limitation of antimicrobics represents an urgent need for both animal and human health, especially for avoiding the spread of resistant bacteria. 

In UTIs, the microscopic evaluation of stained urinary sediment has demonstrated higher diagnostic performance than wet-mount evaluation, for both cytomorphologic identification and bacteria detection [1,2,3]. However, urine culture nowadays represents the gold standard for diagnosing bacterial UTIs in small animals [4] and remains an essential step, as it allows for the determination of antimicrobial resistance rates [5]. Current UTI Guidelines suggest performing urine cultures both for sporadic and recurrent UTIs [5]. Despite this, a positive urine culture result needs to be interpreted with caution, as it is not synonymous with urinary tract infection [5]. Urine culture results should be contextualized with the clinical background of the patient [5]. In fact, a positive urine culture can derive from a true UTI but also from subclinical bacteriuria, or secondary contamination, occurring during collection, conservation, or processing [4]. This important distinction between UTIs and subclinical bacteriuria leads to completely different therapeutic approaches, since only true infections should be treated with antimicrobials [5]. Therefore, for both diagnostic and therapeutic processes, it is crucial to understand which can be the clinical role and the relevance of the bacteriuria during a suspected bladder inflammatory and infective process. For this purpose, it may be useful to associate other easier and cheaper laboratory tests that could provide diagnostic support during the UTI diagnostic process, such as a stained cytologic smear. Cytology can add information to those given by both urine culture and clinical signs, especially with the goal of correctly recognizing the patients that may present active inflammation. In fact, for other biological samples, such as bronchoalveolar lavage and body cavity effusions, morphologic neutrophilic modifications resulting from intracellular bacterial localization are currently employed with the aim of interpreting the role of bacteria throughout an inflammatory process [6,7]. In those cases, finding intracellular bacteria is more likely associated with active infection.

From these observations, the hypothesis of this prospective study is that cytological aspects, such as intracellular bacteria and degenerated neutrophils, as well as clinical signs, may give information that could help to interpret the relevance of urine culture-detected bacteriuria. This study aimed to evaluate the association between the finding of neutrophils and bacteria in unstained and stained urine sediment, and to investigate the potential association between those cytological findings and the presence of clinical signs and positive urine culture.

## 2. Materials and Methods

### 2.1. Study Population and Sample Preparation

A prospective cohort study was conducted using urine samples of dogs referred to the Internal Medicine service of the Veterinary Teaching Hospital “Mario Modenato” of the University of Pisa from March 2020 to December 2021. Dogs with clinically suspected UTIs or with risk factors for UTIs (i.e., endocrinopathies, acute or chronic kidney injury, urolithiasis) were enrolled. 

Dogs presenting one or more urinary clinical signs among dysuria, hematuria, urgency, stranguria, and pollakiuria were included in the symptomatic UTI group [8]. Asymptomatic patients presenting risk factors for UTIs, such as endocrinopathies, acute and chronic kidney injury, and urolithiasis, were also enrolled. For each dog, information about age, sex/sexual status, clinical signs, and ongoing and recent treatments (15 days prior to inclusion) were collected. 

Each dog underwent a physical examination as part of its routine care for its clinical complaint. For each patient, a urinary sample collected by cystocentesis was divided into three aliquots (around 3 mL each): the first aliquot was used for routine urinalysis (chemical analysis dipstick and wet-mount evaluation), the second aliquot was used for stained smear, and the last aliquot was used for urine culture, and antimicrobial susceptibility test. Urine samples were collected through a sterile procedure, and the urine was placed into sterile test tubes and processed for urinalysis within six hours of the collection. However, those designated for urine culture were refrigerated and sent within 24 h to the laboratory for the microbiological processing of the samples [9,10]. Routine urinalysis was performed at the Clinical Pathology Laboratory of the Veterinary Teaching Hospital, while urine cultures and antimicrobial susceptibility tests were performed at the Microbiology Laboratory of the Department of Veterinary Sciences of the University of Pisa. Routine urinalysis included physical observations, urine-specific gravity tests using an optic refractometer (Rhino VET 360 Reichert), chemical analysis (Idexx VetLab UA analyser with Idexx UA strips), and wet-mount microscopic evaluations performed by non-boarded veterinarians, with more than 20 years of experience. 

The remaining quote was centrifugated at room temperature for 5 min at 1000–1500 rpm. The supernatant was removed, leaving approximately 2–3 urine drops; then, the sediment was resuspended by gentle shaking; one drop was placed on a slide, covered with a coverslip, and used for wet-mount microscopic evaluation [10]. 

The reporting system for wet-mount evaluation included several parameters. Each of the following parameters was quantitatively indicated based on the mean observed in 10 fields at 10× magnification: epithelial cells (10×); casts (10×); crystals (10×); parasites (10×); bacteria (1+ to 5+ grading based on mean 10 fields 10× [10]; red blood cells (10×); leucocytes (10×).

**Figure 1:**
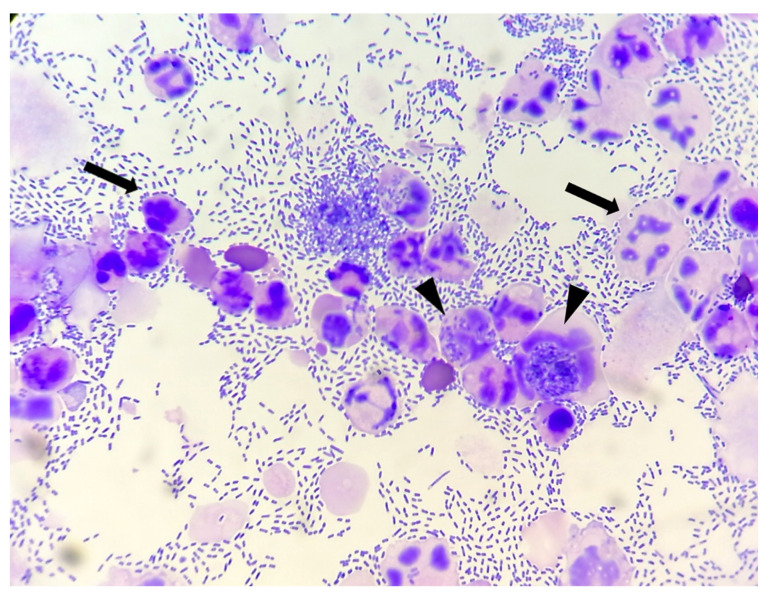
Canine urinary cytology performed after cytocentrifugation. Numerous rods are present, both extracellular and intracellular (arrow heads). Degenerated neutrophils are also visible (arrows). (100× Oil).

**Figure 2:**
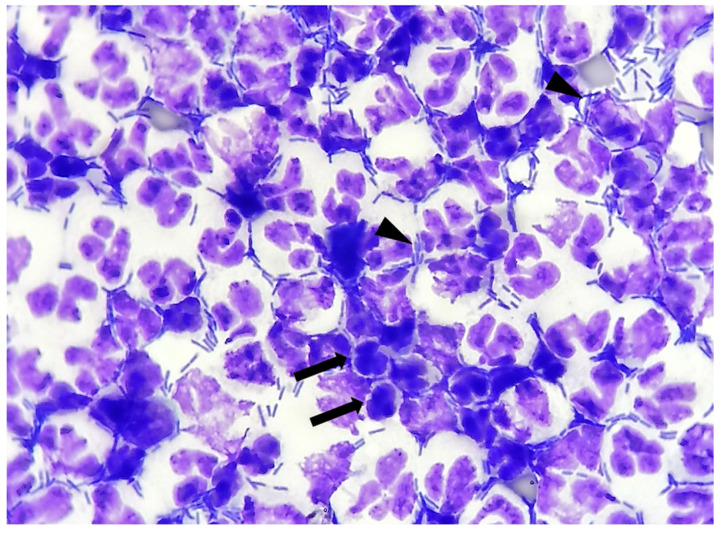
Canine urinary cytology performed after cytocentrifugation. Numerous neutrophilic degeneration details (arrows): nuclear swelling, karyolysis, pyknosis. Numerous rods are present, both extracellular and intracellular (arrow heads). (100× Oil).

To increase slide cellularity, preserving as much cellular morphology as possible [11], the aliquots for stained cytological evaluation underwent cytocentrifugation (Wescor). For each dog, 1–2 slides were prepared, manually stained through a rapid Romanowsky stain (MGG QUICK STAIN—Bio Optica), and blindly evaluated by a cytopathologist for both clinical and laboratory information. In each cytological smear the following findings were recorded:

- Bacteria: indicated as present/absent, morphologically identified as rods/cocci/filamentous, and quantified with an estimate of 10 fields at 100× magnification in occasional <3 bacteria/100×, few 3–10 bacteria/100×, moderate 11–40 bacteria/100×, and many >40 bacteria/100× [12]. When detected, bacteria were also classified as intracellular and/or extracellular (Figure 1).
- Neutrophils: indicated as present/absent
- Degenerate neutrophils: indicated as present/absent. Degeneration was defined if karyolysis (swollen, pale-staining nuclei) or nuclear modifications, like swelling or vacuoles occupying much of the cell [13] (Figure 2).

### 2.2. Microbiological Analysis

Aerobic urine culture results were obtained from both a qualitative and quantitative point of view, considering bacterial species, bacterial count, and antimicrobial susceptibility patterns observed for each isolated uropathogen. The number of colony-forming units/milliliter (CFU/mL) of urine and the involved bacterial strains were determined for each sample, following the protocol previously reported by Papini et al. (2006) [14]. Samples with ≥100 CFU/mL were considered positive. The in vitro antibiotic sensitivity test was performed for each bacterial isolate by using the disc diffusion method, as reported by the Clinical and Laboratory Standards Institute (CLSI) [15]. The following antibiotics (Oxoid) were employed: amikacin (30 µg), amoxicillin–clavulanic acid (20 + 10 µg), ampicillin (10 µg), azithromycin (15 µg), cefovecin (30 µg), cefazolin (30 µg), ceftriaxone (30 µg), clindamycin (2 µg), doxycycline (30 µg), enrofloxacin (5 µg), marbofloxacin (5 µg). The results were interpreted as indicated by EUCAST [16].

### 2.3. Statistical Analysis

Statistical analysis was performed using the software SPSS Statistics (IBM Corp., New York, NY, USA). Age, urinary pH, and the urine’s specific gravity were evaluated as continuous variables and, as non-parametric, expressed using the median and minimum-maximum range. Difference in age between symptomatic/asymptomatic dogs was investigated using the Mann–Whitney test. 

All other variables, such as presence/absence of specific clinical signs, dipstick parameters (proteins, glucose, bilirubin, urobilinogen, leukocytes, blood), presence/absence of bacteria at wet-mount evaluation (from 1+ to 5+), presence/absence of bacteria at stained cytological evaluation (both intracellular and/or extracellular), bacterial typology, presence/absence of neutrophils and degenerated neutrophils, and positive/negative urine culture were analyzed as categorical variables. Differences in dipstick analysis between the symptomatic and asymptomatic group were investigated using the Chi-square test (in case of *n* = 0, categories were unified to permit the statistical analysis). Sensibility, specificity, and positive/negative predictive values of wet-mount against cytologically-detected bacteriuria were estimated.

Associations between urine culture and cytologic findings (bacteria, neutrophils, intracellular bacteria, degenerated neutrophils) were evaluated using the Chi-square test. Respective Odds Ratios were calculated. Afterward, multivariate backward stepwise binary logistic regression was performed to assess the association of variables that resulted in significant associations (clinical signs, degenerated neutrophils, intracellular bacteria) with urine culture outcome in the univariate analysis. Associations between clinical signs and intracellular bacteria were investigated using the chi-square test. Differences in total bacterial count between patients with or without clinical signs were investigated through the Mann–Whitney test after the normality Kolmogorov–Smirnov test. Additionally, *p* values < 0.05 were considered statistically significant in all the tests.

## 3. Results

### 3.1. Signalment

Ninety dogs were prospectively enrolled. Various breeds were represented: Dachshund (*n* = 7), Golden Retriever (*n* = 6), German Shepherd (*n* = 5), Jack Russel Terrier (*n* = 4), Bernese Mountain Dog (*n* = 3), Dobermann (*n* = 3), English Bulldog (*n* = 3), Basset Hound (*n* = 2), Bobtail (*n* = 2), Boxer (*n* = 2), Border Collie (*n* = 2), Cavalier King Charles Spaniel (*n* = 2), Swiss Mountain Dog (*n* = 2), Cocker Spaniel (*n* = 2), Shih Tzu (*n* = 2), Maltese (*n* = 2), Shar Pei (*n* = 2), Australian Shepherd (*n* = 1), Galgo (*n* = 1), Cane Corso (*n* = 1), Breton (*n* = 1) Husky Siberian (*n* = 1), Kurzhaar (*n* = 1), Maremma Sheepdog (*n* = 1), Schnauzer (*n* = 1), English Setter (*n* = 1), Toy Poodle (*n* = 1), German Spitz (*n* = 1), Springer Spaniel (*n* = 1), Newfoundland Dog (*n* = 1). Twenty-six dogs were mixed-breed. Median age was 8.25 years (range 0.4–16 years). Forty-three (47.8%) were female (22 spayed and 21 intact), and forty-seven (52.2%) were male (5 neutered and 42 intact). 

Nineteen dogs were diagnosed with UTIs according to ISCAID guidelines [5], whereas the remaining 19 dogs with positive urine cultures were diagnosed with subclinical bacteriuria.

Thirty-five out of ninety (38.9%) dogs were symptomatic at the time of the inclusion, whereas the remaining 55 patients (61.1%) were considered asymptomatic. Among symptomatic dogs, 22 of 35 (62.9%) were male, and the remaining 13 (37.1%) were female. Otherwise, among asymptomatic dogs, 25 out of 55 (45.4%) were male, and the remaining 30 (54.6) were female. The median age in the symptomatic group was nine years (range 0.4–16), and in asymptomatic group it was 8.1 years (range 0.5–15.2). There was not a statistically significant difference in age between symptomatic and asymptomatic dogs (*p* value = 0.6253).

### 3.2. Physical and Chemical Urinalysis

Urinary specific gravity had a median value of 1017 (range 1002–1055). The median values of urinary pH and urine protein/creatinine ratio (UP:UC) were 7 (range 5.0–9.0) and 1.38 (range 0.01–41.33), respectively. One sample (1.1%) of the 90 submitted for chemical analysis could not be analyzed due to the intense degree of hematuria. Results of the chemical analysis are reported in Table 1. 

### 3.3. Microbiological Evaluation

Thirty-eight out of 90 (42.2%) samples had a positive urine culture. Bacterial isolates, total bacterial counts (TBC) expressed as colony-forming units per milliliter (CFU/mL), and antimicrobial sensitivity test results are reported in Appendix A. Twenty-five (65.8%) isolated bacterial strains out of 38 were resistant to three or more categories/molecules of the following antimicrobics: third generation cephalosporins (cefovecin, ceftriaxone), first generation cephalosporins (cefazolin), fluoroquinolones (enrofloxacin, marbofloxacin), amoxicillin clavulanate, ampicillin, azithromycin, amikacin, clindamycin, and doxycycline.

### 3.4. Wet-Mount and Cytological Stained Evaluation

Wet-mount examination showed bacteria in 32 dogs (35.6%): five (5.6%) samples were scored 1+, seven (7.8%) 2+, 11 (12.2%) 3+, and nine (10%) 4+. No bacteria were found in the remaining 58 samples (64.4%). 

In stained urine samples, bacteria were identified in 38 dogs (42.2%). Among these, six dogs had rare bacteria (6.7%), 11 dogs had few bacteria (12.2%), 12 dogs had moderate bacteria (13.3%), and 12 dogs had many bacteria (13.3%). The remaining 52 patients (57.8%) did not show bacteria in stained urine samples. Differences between wet-mount and cytologic stained smear evaluation in terms of sensibility, specificity, and positive/negative predictive values, considering urine culture as the gold standard, are reported in Table 2. 

Both wet-mount bacteriuria and the cytological presence of intracellular and extracellular bacteria, neutrophils, and degenerated neutrophils were successively associated with positive urine culture (Table 3).

In the multivariate analysis (Table 4) the only independent predictor for positive urine culture was the presence of intracellular bacteria (OR = 23.073 95% CI 7.246–73.471; *p* < 0.0001). 

**Figure 3:**
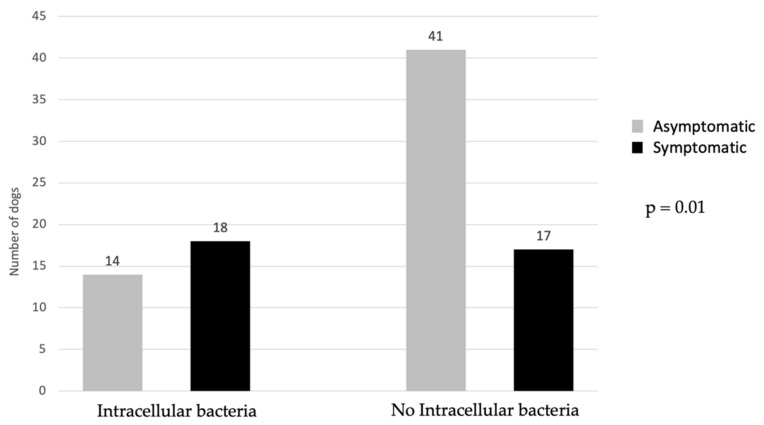
Comparison of symptomatic and asymptomatic dogs in regard to presence/absence of intracellular bacteria. Applied statistical test: Pearson Chi-Square test.

**Figure 4:**
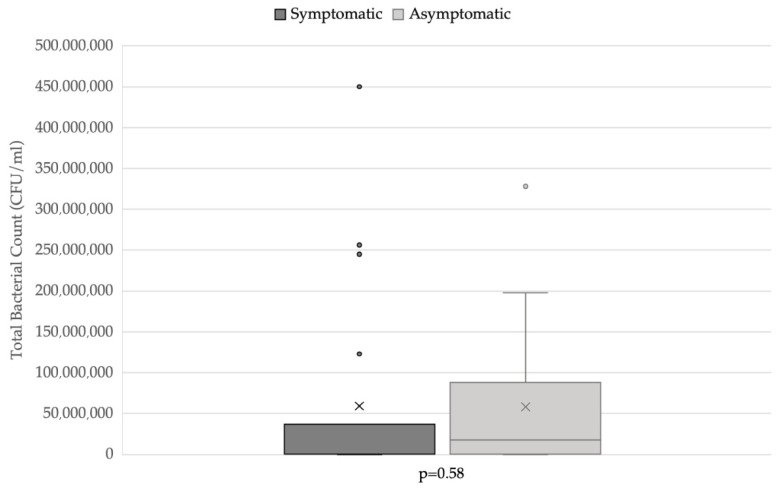
Total bacterial count distribution in symptomatic and asymptomatic dogs. Applied statistical test: Mann-Whitney test.

Of the patients, 50% with positive urine culture showed clinical signs, while 56.3% of patients with intracellular bacteria were symptomatic. The prevalence of symptomatic and asymptomatic patients in relation to the presence of intracellular bacteria is reported in Figure 3. Total bacterial count did not differ significantly between symptomatic (*n* = 19) and asymptomatic (*n* = 19) dogs (Figure 4).

## 4. Discussion

In agreement with previous reports, the most frequently isolated bacterial strains in our study were Enterobacteriaceae, mainly *E. coli*. Twenty-five (65.8%) isolated bacterial strains of 38 were resistant to three or more antimicrobics categories/molecules. This result traced the European antibiotic-resistance patterns found among urinary pathogens [17], indicating the urgent need to limit the use of antibiotics only when strictly necessary, based on clinical signs, urine culture, and antibiotic sensitivity tests.

Regarding dipstick urinalysis, leukocytes and urobilinogen seemed to be significatively lower in asymptomatic dogs. However, both the dipstick pads for leukocytes and urobilinogen are not considered accurate in veterinary species [18,19].

Regarding the detection of bacteria in urine sediment, previous studies reported higher sensibility and specificity [1,2] when different stains were used. It is reasonable to suppose that numerous urine amorphous particles, visible at wet-mount sediment evaluation, may create difficulties for the identification of bacteria, potentially reducing both sensibility and specificity. Therefore, sensibility and specificity in detecting bacteriuria represent critical points of wet-mount evaluation. In our study, stain implementation did not increase specificity, but it did increase sensibility. Each sample underwent cytocentrifugation. Therefore, it is difficult to understand if and how much the cytocentrifugation process might contribute to the increased sensibility of stained cytologic evaluation. 

According to our results, the detection of bacteriuria during wet-mount evaluation increased the probability of presenting a positive urine culture 7-fold. This probability became higher when bacteria were searched on urine sediment-stained smears. In fact, patients presenting cytologically evident bacteriuria showed a 20-fold chance of having a positive urine culture. However, if intracellular bacteria were seen, the probability of a positive urine culture increased by 23-fold. As the finding of intracellular bacteria is commonly considered a marker of active inflammation, it is plausible that patients showing intracellular bacteria had a higher probability of showing a greater bacterial count or of vital bacteria, able to grow significantly in vitro, thus causing positive urine cultures. According to our results, if intracellular bacteria were observed in cytologic stained smears, the probability of presenting a positive urine culture increased significantly.

Curiously, five patients presented a negative urine culture, while intracellular bacteria and degenerated neutrophils were identified at the cytologic exam. Three out of five of these dogs contextually presented clinical signs consistent with UTIs. A false-negative urine culture may derive from bacterial destruction or neutralization during sample conservation or prior to microbiologic examination [20]. False negative results may also be a consequence of hyposthenuria, which may cause bacterial dilution [21]. These findings could suggest a potential key role of stained cytology in helping clinicians in the diagnostic process and the management of symptomatic patients presenting a negative urine culture.

Although the patients in our study had a 6-fold chance to show positive urine culture if leukocyturia was noticed, not all patients with pyuria had positive urine cultures. The discrepancy between pyuria and bacteriuria is not surprising, as a previous study reported that these findings might not be simultaneous and differently associated with positive urine culture [1]. This finding agrees with the assertion that bacteriuria and UTI are not synonymous, and that the diagnosis of a UTI implies the presence of clinical signs or the host’s inflammatory response. 

In the present study, subclinical bacteriuria represented a conspicuous part of enrolled cases, while only 42.1% of dogs with positive urine cultures showed clinical signs of UTI. The elevated prevalence of bacteriuria may be secondary to urine sampling. Although cystocentesis is a sterile procedure, it is not possible to exclude some degree of contamination during sampling [10,22]. When the sample is collected by cystocentesis, lower TBC (<10^3^ CFU) can be due to contamination; however, any amount of bacteria can be considered responsible for UTIs [5]. Thus, considering the possibility of secondary contamination, stained cytologic evaluation may be a useful diagnostic tool for understanding the clinical significance of a urine culture-detected bacteriuria by evaluating the presence/absence of cytologic inflammatory aspects. As expected, the presence of degenerated neutrophils was mainly associated with active or highly recent infections and showed a 12-fold probability of positive urine culture.

In our study bacteria phagocytosis was always observed in association with neutrophil degeneration. This finding agreed with the cytological association between the phagocytosis process and cellular degeneration [23,24]. Multivariate analysis showed that the presence of intracellular bacteria could predict a positive urine culture outcome (HR = 23,073 95% CI 7.246–73.471; *p* < 0.0001). This result may encourage investigation into a potential synergy of the concomitant use of cytological stained smear evaluation of urine sediment and urine culture.

Like results found by Bartges in 2004 [4], only the 42.1% (16/38) of the patients with positive cultures were symptomatic, whereas the 30.8% of the patients with negative cultures showed clinical signs at the time the sample was collected. These results clearly displayed how difficult it may be to set up the therapeutic approach on the presence of clinical signs. 

Assuming that the compresence of intracellular bacteria and degenerated neutrophils could be reasonably associated with an active septic inflammation, we aimed to investigate how these findings might be associated with typical clinical signs of UTIs. Among patients with a positive urine culture, 42.1% were symptomatic, while among those presenting intracellular bacteria, 56.3% were symptomatic. This finding seemed to suggest that cytologic signs of active inflammation and infection may be associated with a diagnosis of UTIs better than positive urine culture alone. This result could suggest that, as for other biological samples [6,7], intracellular bacteria could be more likely associated with active bacterial infection and inflammation, thus resulting in true UTIs. 

The cytologic evaluation of these parameters may support, together with clinical information, the correct interpretation of urine culture results, especially when the urine culture, urinalysis, and clinical aspects disagree. In these cases, the cytologic evaluation of urinary sediment may provide additional information, which may suggest the use of further investigation or diagnostic techniques, such as cystoscopy, a biopsy of the bladder mucosa, and/or bladder culture. Lastly, TBC was not higher in symptomatic patients; this finding seemed to confirm that subclinical bacteriuria could also be associated with a large number of isolated microorganisms [5,25]. Therefore, the presence of a high TBC alone should not lead to the administration of antimicrobics.

Our study had some limitations. Although history collection was standardized, the number of symptomatic patients might be underestimated in the case of mild and undetected clinical signs. During this study, we did not register the clinical, clinicopathological, and microbiological follow-up of patients; it could be interesting to evaluate the clinical outcome of patients in relation to the therapeutic protocol. Nowadays, there is no evidence that both canine and feline patients with pyuria and bacteriuria and not treated with antibiotics had the worst outcomes, even if comorbidities, such as diabetes or CKD, are present [26,27,28]. Nevertheless, it could be of great interest to investigate the potential association between the presence of intracellular bacteria and the clinical outcome in treated and untreated patients. 

## 5. Conclusions

Urine culture should be interpreted based on clinical signs, as the presence of bacteria in urine does not always have clinical implications. In this context, cytologic stained evaluation of urine sediment may have good feasibility, adding useful information during the diagnostic process. Cytologic evaluation, together with the specific research of intracellular bacteria, may suggest the presence of an active bacterial inflammatory process, therefore being useful in supporting the decisional process for antibiotic administration. Additional research on intracellular bacteria may strengthen the evidence of active bacterial inflammation or not and help to avoid unnecessary empirical antimicrobial treatment while waiting for urine cultures and antimicrobial sensitivity test results. Moreover, as the presence of pyuria and/or bacteriuria are not synonyms with UTIs, detection of intracellular bacteria in patients with negative urine cultures or presenting as asymptomatic, may suggest the need to re-examine the case from both a clinical and microbiological point of view. However, the presence of active cytological inflammation associated to clinical signs and positive urine culture may strengthen the evidence regarding treatment need.

## Figures and Tables

**Table 1:** Chemical urinalysis results (Dipstick evaluation).

Dipstick Chemical Analysis					
Parameter	Quantitative Estimation	Number of Samples (%)	N Symptomatic (%)	N Asymptomatic (%)	*p* Value *
Leucocytes	Absent	49 (55)	11 (22.5)	38 (77.5)	**0.0027**
	25 leucocytes/μL	13 (14.6)	9 (69)	4 (31)	
	100 leucocytes/μL	7 (7.9)	5 (71.4)	2 (28.6)	
	500 leucocytes/μL	20 (22.5)	9 (45)	11 (55)	
Proteins	Absent	44 (49.4)	18 (41)	26 (59)	0.9307
	30 mg/dL	24 (27)	11 (46)	13 (54)	
	100 mg/dL	8 (9)	4 (50)	4 (50)	
	500 mg/dL	13 (14.6)	4 (31)	9 (69)	
Glucose	Absent	79 (88.8)	32 (40.5)	47 (59.5)	0.6736
	50 mg/dL	2 (2.2)	0 (0)	2 (100)	
	300 mg/dL	3 (3.4)	1 (33.3)	2 (66.7)	
	1000 mg/dL	5 (5.6)	1 (20)	4 (80)	
Ketones	Absent	80 (89.9)	30 (37.5)	50 (62.5)	0.7271
	15 mg/dL	7 (7.9)	4 (57)	3 (43)	
	50 mg/dL	2 (2.2)	0 (0)	2 (100)	
Urobilinogen	Normal	76 (85.4)	25 (33)	51 (67)	**0.0187**
	1 mg/dL	7 (7.9)	6 (85.7)	1 (14.3)	
	4 mg/dL	5 (5.6)	2 (40)	3 (60)	
	8 mg/dL	1 (1.1)	1 (100)	0 (0)	
Bilirubin	Absent	76 (85.5)	26 (34.2)	50 (65.8)	0.1624
	1 mg/dL	6 (6.7)	4 (66.7)	2 (33.3)	
	3 mg/dL	6 (6.7)	3 (50)	3 (50)	
	6 mg/dL	1 (1.1)	1 (100)	0 (0)	
Blood	Absent	28 (31.5)	9 (32)	19 (68)	0.2669
	10 erythrocytes/μL	9 (10.1)	5 (55.5)	4 (44.5)	
	25 erythrocytes/μL	15 (16.8)	6 (40)	9 (60)	
	50 erythrocytes/μL	9 (10.1)	1 (11.1)	8 (88.9)	
	250 erythrocytes/μL	28 (31.5)	13 (46.4)	15 (53.6)	

* *p* values refer to the comparison between symptomatic and asymptomatic dogs. Applied statistical test: Pearson Chi-Square test.

**Table 2:** Comparison between wet-mount evaluation and stained cytology.

Parameter	Bacteria with Wet-Mount Evaluation		Bacteria with Stained Cytology (Diff-Quick)	
	Value	95% CI	Value	95% CI
Sensibility	61%	0.45–0.74	76%	0.61–0.87
Specificity	83%	0.70–0.91	83%	0.70–0.91
Positive Predictive Value	**72%**	0.65–0.79	**76%**	0.69–0.83
Negative Predictive Value	74%	0.68–0.80	**83%**	0.78–0.88
*p* value	<0.0001		<0.0001	

Comparison of sensibility, specificity, and positive/negative predictive values in diagnosing a positive urine culture between wet-mount evaluation (left) and in Diff-Quick stained cytocentrifugated samples (right). Applied statistical test: Pearson Chi-Square test.

**Table 3:** Association between wet-mount and stained cytologic findings with positive urine culture outcome.

Finding	Positive UC (n = 38)	Negative UC (n= 52)	Total	OR	CI 95%	*p* Value
Bacteria (WM)	23 (60.5%)	9 (17.3%)	32 (28.8%)	**7.1**	2.77–19.31	<0.001
Extracellular bacteria (SC)	30 (78.9%)	8 (15.4%)	38 (42.2%)	**20.6**	6.97–60.99	<0.001
Intracellular bacteria (SC)	27 (71%)	5 (9.6%)	32 (35.6%)	**23.1**	7.24–73.47	<0.001
Neutrophils (SC)	29 (76.31%)	17 (32.7%)	46 (51.1%)	**6.6**	2.57–17.08	<0.001
Degenerated neutrophils (SC)	29 (76.31%)	11 (21.1%)	40 (44.4%)	**12**	4.41–32.68	<0.001
Intracellular bacteria and degenerated neutrophils (SC)	27 (71%)	5 (9.6%)	32 (35.6%)	**23.1**	7.24–73.47	<0.001

WM: wet-mount; SC: stained cytology; UC: urine culture.

**Table 4:** Multivariate analysis of predictors of positive urine culture.

Phase		Sig.	OR	Lower 95% OR	Upper 96% OR
Phase 1	SCS	0.647	1.32	0.406	4.26
	IB	0.008	13.97	1.969	99.18
	DN	0.576	1.67	0.274	10.26
Phase 2	IB	0.003	16.20	−2.514	104.4
	DN	0.641	1.52	0.262	8.78
Phase 3	IB	**0.000**	**23.07**	7.246	73.47

Results of the multivariable backward stepwise binary logistic regression model for positive urine culture outcome. SCS: specific clinical signs; IB: intracellular bacteria; DN: degenerated neutrophils; Sig: statistical significance; OR: odds ratio.

## Data Availability

Not applicable.

## Appendix A

**Table A1:** Bacterial isolates, TBC, and antimicrobial sensitivity test results.

Isolate	Bacteria	TBC (CFU/mL)	AM	A\C	CV	CZ	CX	EF	MF	AZ	AK	CL	DX
1	Other Enterobacteriaceae	1.2 × 10^2^	I	S	NT	NT	NT	S	NT	S	S	R	I
2	Other Enterobacteriaceae	2.5 × 10^8^	R	R	NT	NT	NT	R	NT	NT	NT	R	R
3	Other Enterobacteriaceae	2.5 × 10^8^	R	R	NT	NT	NT	I	NT	R	S	R	R
4	Other Enterobacteriaceae	5.1 × 10^7^	R	R	NT	NT	NT	S	NT	S	S	R	R
5	Other Enterobacteriaceae	3 × 10^2^	R	R	S	R	S	S	S	S	S	R	S
6	*Escherichia coli*	1.7 × 10^7^	R	I	NT	NT	NT	S	NT	S	S	R	S
7	*Escherichia coli*	6 × 10^5^	S	S	S	NT	NT	S	S	NT	S	NT	S
8	*Escherichia coli*	3.3 × 10^8^	R	I	I	S	S	S	S	R	I	R	S
9	*Escherichia coli*	6 × 10^5^	R	S	S	NT	NT	S	S	NT	S	NT	S
10	*Escherichia coli*	6.5 × 10^7^	R	I	NT	NT	NT	R	NT	R	I	R	I
11	*Escherichia coli*	2.9 × 10^4^	R	I	I	S	I	S	S	R	R	R	S
12	*Escherichia coli*	2.7 × 10^7^	R	S	NT	NT	NT	S	NT	S	S	R	S
13	*Escherichia coli*	1.9 × 10^8^	R	R	NT	NT	NT	S	NT	NT	NT	R	I
14	*Escherichia coli*	2.5 × 10^8^	R	R	NT	NT	NT	R	NT	S	S	R	S
15	*Escherichia coli*	8.8 × 10^7^	R	I	I	S	S	I	I	R	S	R	S
16	*Escherichia coli*	1 × 10^8^	R	I	NT	NT	NT	S	NT	S	S	R	S
17	*Escherichia coli*	2.9 × 10^6^	I	S	S	S	S	S	S	R	I	R	S
18	*Escherichia coli*	4.2 × 10^5^	R	S	S	S	S	S	S	R	I	R	I
19	*Escherichia coli*	6 × 10^5^	S	S	S	NT	NT	S	S	NT	S	NT	S
20	*Escherichia coli*	2.3 × 10^6^	R	I	S	S	S	I	S	I	R	R	S
21	*Escherichia coli*	1.56 × 10^4^	R	I	R	R	R	R	R	S	S	R	S
22	*Escherichia coli*	1.23 × 10^8^	I	I	I	S	S	S	I	I	I	R	S
23	*Escherichia coli*	1.03 × 10^3^	S	I	R	S	S	S	S	S	S	R	S
24	*Escherichia coli*	3.7 × 10^7^	R	I	I	S	S	S	S	R	S	R	R
25	*Klebsiella* spp.	6 × 10^5^	R	S	R	R	R	R	R	NT	S	R	S
26	*Klebsiella* spp.	1.3 × 10^5^	R	R	R	NT	R	R	R	S	R	R	R
27	*Klebsiella* spp.	6 × 10^5^	R	R	R	R	R	R	R	R	S	R	S
28	*Klebsiella* spp.	4.5 × 10^8^	R	I	R	R	R	R	R	S	S	R	S
29	*Proteus* spp.	6 × 10^5^	S	S	S	S	S	S	S	S	S	S	R
30	*Pseudomonas* spp.	2.9 × 10^5^	R	R	NT	NT	NT	R	NT	NT	S	R	R
31	*Pseudomonas* spp.	4.6 × 10^5^	R	R	R	R	R	R	R	R	S	R	S
32	*Pseudomonas* spp.	5.4 × 10^2^	R	R	NT	NT	NT	I	NT	R	S	R	I
33	*Pseudomonas* spp.	3.7 × 10^7^	R	R	NT	NT	NT	I	NT	I	S	R	R
34	*Staphylococcus* spp.	6 × 10^5^	S	S	S	NT	NT	S	S	NT	NT	S	S
35	*Staphylococcus* spp.	6.5 × 10^4^	R	R	NT	NT	NT	R	NT	R	S	R	R
36	*Staphylococcus* spp.	5.8 × 10^4^	R	S	R	S	R	R	R	R	R	R	R
37	*Staphylococcus* spp.	3.2 × 10^6^	R	S	S	S	S	S	NT	S	S	I	S
38	*Streptococcus* spp.	1.2 × 10^2^	S	S	S	S	S	I	I	R	R	R	R

AP, ampicillin; AC, amoxicillin clavulanate; CV, cefovecin; CZ cefazolin; CX, ceftriaxone; EF, enrofloxacin; MF, marbofloxacin; AZ, azithromycin; AK, amikacin; CL, clindamycin; DX, doxycycline. S, Sensitive; I: intermediate; R, Resistant; NT, Non-tested.
